# Effect of laparoscopic sleeve gastrectomy on male reproductive function in Chinese men with obesity: a prospective cohort study

**DOI:** 10.1097/JS9.0000000000001328

**Published:** 2024-03-12

**Authors:** Xiang Gao, Pengzhou Li, Guohui Wang, Weizheng Li, Zhi Song, Shaihong Zhu, Liyong Zhu

**Affiliations:** Department of Metabolic and Bariatric Surgery, The Third Xiangya Hospital of Central South University, Changsha, People’s Republic of China

**Keywords:** erectile function, laparoscopic sleeve gastrectomy, obesity, semen quality

## Abstract

**Background:**

Obesity is a widely recognized global public health issue, and bariatric surgery has emerged as an effective intervention for alleviating obesity associated health complications. However, the impact of bariatric surgery on male reproductive function remains inconclusive in the literature. The current understanding of the impact of laparoscopic sleeve gastrectomy (LSG) on male reproductive function remains ambiguous, despite its status as the most commonly performed bariatric surgery. This prospective cohort study aimed to investigate the impact of LSG on erectile function and semen quality.

**Patients and methods:**

A total of 34 obese patients were enrolled in this study and underwent LSG. Prior to the operation and at 3, 6, and 12 months postoperation, all participants were required to complete the International Index of Erectile Function-5 (IIEF-5) questionnaire and undergo a nocturnal erectile function test and semen quality analysis.

**Results:**

Within 12 months postoperation, BMI, blood lipids, and insulin resistance showed significant improvement. The IIEF-5 score increased significantly (18.88±5.97 vs. 23.78±3.19, *P*<0.05), and the frequency and duration of erections significantly improved compared to baseline. Sperm concentration, total motility, survival rate, and sperm morphology parameters exhibited a significant decline at 3 months but demonstrated a significant improvement at 6 and 12 months postoperation. At 12 months, sperm concentration was shown to be correlated with changes in zinc (r=0.25, *P*=0.033) as well as changes in testosterone (r=0.43, *P*=0.013).

**Conclusions:**

LSG has beneficial effects on erectile function, despite a transient decline in semen quality at 3 months postoperatively, followed by a significant improvement at 12 months.

## Introduction

Highlights

- Laparoscopic sleeve gastrectomy (LSG) improves erectile function in obese men, indicating its positive impact on male reproductive function.
- Semen quality decreases at 3 months post-LSG but improves significantly at 12 months.
- Changes in zinc and testosterone concentrations correlate with sperm concentration at 12 months postoperatively.
- This study provides insights into the effects of LSG on male reproductive function, including improved erectile function and changes in semen quality and hormonal levels.

Obesity and its associated metabolic diseases have emerged as chronic noncommunicable diseases that pose a significant threat to human health, constituting a global public health issue^1,2^. According to data from the China National Nutrition and Health Survey, there has been a rapid increase in overweight and obesity rates, with the latest prevalence estimates for adults from 2015 to 2019, based on Chinese criteria, indicating that ~34.3% of adults are overweight and 16.4% are classified as obese^3^. Obesity not only elevates the risk of metabolic diseases such as hypertension, type 2 diabetes, cardiovascular diseases, and malignant tumors^4,5^ but also exerts an impact on reproductive function^6,7^.

In men, obesity can increase the risk of male obesity–secondary hypogonadism (MOSH), which can have various effects on male fertility, including semen quality, sperm DNA integrity, and erectile function^8^. Currently, there is a limited number of prospective studies examining the impact of bariatric surgery on male reproductive function, particularly within the Chinese population. The most recent retrospective study investigating the effects of bariatric surgery on reproductive function in obese Chinese men was conducted in 2015 by Kun *et al*.^9^. However, that study solely focused on changes in erectile function and did not consider alterations in semen quality. Consequently, the implications of changes in semen quality remain a pressing clinical concern that requires attention.

The impact of bariatric surgery on male reproductive function has been a subject of debate^10,11^. A case series conducted by Di Frega *et al*.^12^ in 2005 reported postoperative nonobstructive azoospermia, complete cessation of spermatogenesis, and normal sex hormone levels in six Roux-en-Y gastric bypass (RYGB) patients. In 2012, Sermondade *et al*.^13^ reported three patients who experienced significant deterioration in semen parameters following bariatric surgery, including severe asthenozoospermia and teratozoospermia, but no cases of azoospermia were observed. Additionally, a prospective study conducted in 2017 indicated that only semen volume and sperm motility showed significant improvement 6 months after RYGB^14^. Overall, there are conflicting viewpoints among scholars, with some suggesting that the quality of semen significantly improves after RYGB, while others contend that it significantly worsens.

Both RYGB and laparoscopic sleeve gastrectomy (LSG) are prominent bariatric surgery procedures. Currently, LSG has gained global popularity and has surpassed other methods as the most prevalent bariatric surgery approach. Unlike RYGB, LSG does not involve altering the normal intestinal anatomy but instead reduces the size of the gastric pouch, thereby restricting food intake. However, the impact of LSG on semen quality and erectile function in obese men remains unclear. In clinical practice, male patients express significant concern regarding this issue, making it clinically significant to investigate. Therefore, the objective of this study was to assess the effects of LSG on male reproductive function in Chinese patients with obesity.

## Patients and methods

This work, which has been reported in line with the strengthening the reporting of cohort, cross-sectional, and case–control studies in surgery (STROCSS) criteria^15^, included a prospective cohort data collection of eligible patients with obesity who underwent LSG between January 2020 and December 2021. All included patients signed informed consent forms at the first appointment. This study was approved by the local ethics committee and institutional review board and is registered on clinicaltrials.gov. The patients were all from the Bariatric and Metabolic Surgery Center of Hospital A, which is where they underwent semen quality analysis, nocturnal penile tumescence (NPT) monitoring, and seminal trace element analysis.

### Patients

All patients were included from January 2020 to December 2021 and underwent the same preoperative evaluation. According to the latest guidelines released by Chinese Society for Metabolic and Bariatric Surgery in 2019, bariatric surgery is strongly recommended for patients with a BMI >32.5 kg/m^2^. Conversely, for patients with a BMI <32.5 kg/m^2^, bariatric surgery might be considered only when lifestyle changes and medical therapy fail to control the condition, and at least two components of the metabolic syndrome are present or complications are observed. Therefore, to eliminate potential confounding factors, this study included a BMI cut-off of 32.5 kg/m^2^. The inclusion criteria were as follows: 1) male patients aged 20–35 years; 2) BMI ≥32.5 kg/m^2^ and meeting the indications for metabolic surgery; 3) willing and able to adhere to the follow-up protocol and study procedures; and 4) able to understand and sign the informed consent form for research and, if illiterate, willing to put fingerprints on the informed consent form. The exclusion criteria were as follows: 1) a history of drug addiction, smoking (> two cigarettes/week), and alcohol drinking (>2 times/month); 2) mental illnesses that may affect compliance with clinical studies, including dementia, active psychosis, major depression, or attempted suicide; 3) scrotal injury or previous scrotal surgery; 4) use of a phosphodiesterase-5 inhibitor; 5) structural abnormalities in the penis; and 6) engagement in chemical production or electromagnetic radiation-related industries for a long time.

Fasting insulin, fasting blood glucose, homeostasis model assessment of insulin resistance (HOMA-IR), and BMI were collected before and 3, 6, and 12 months after surgery. HOMA-IR was calculated by the following formula: HOMA-IR = fasting insulin (μIU/l) × fasting glucose (mmol/l)/22.5, which has been verified to correlate well with the euglycemic clamp^16^.

### Demographic characteristics

Demographic data such as age, marital status, and educational level were collected via a questionnaire. The Health History Checklist for patients was used to assess the patient’s health condition. This checklist included the following items: (1) whether the participants had been currently diagnosed with specific disorders (e.g. hypertension, diabetes, dyslipidemia, and metabolic syndrome); (2) whether they had experienced spinal cord injury, radical pelvic surgery, scrotal trauma, cavernous fibrosis, or prostatic disease; and (3) whether they had taken phosphodiesterase type 5 inhibitors or any other hormone replacement therapy. Finally, the frequency of alcohol consumption and smoking was recorded.

### Erectile function evaluation

The International Index of Erectile Function-5 (IIEF-5)^17^ questionnaire was completed by the patients before and t3 and 6 months after surgery. An IIEF-5 score less than 7 points indicates severe erectile dysfunction (ED), 8–11 points indicates moderate ED, and 12–21 points indicates mild ED.

**Figure 1:**
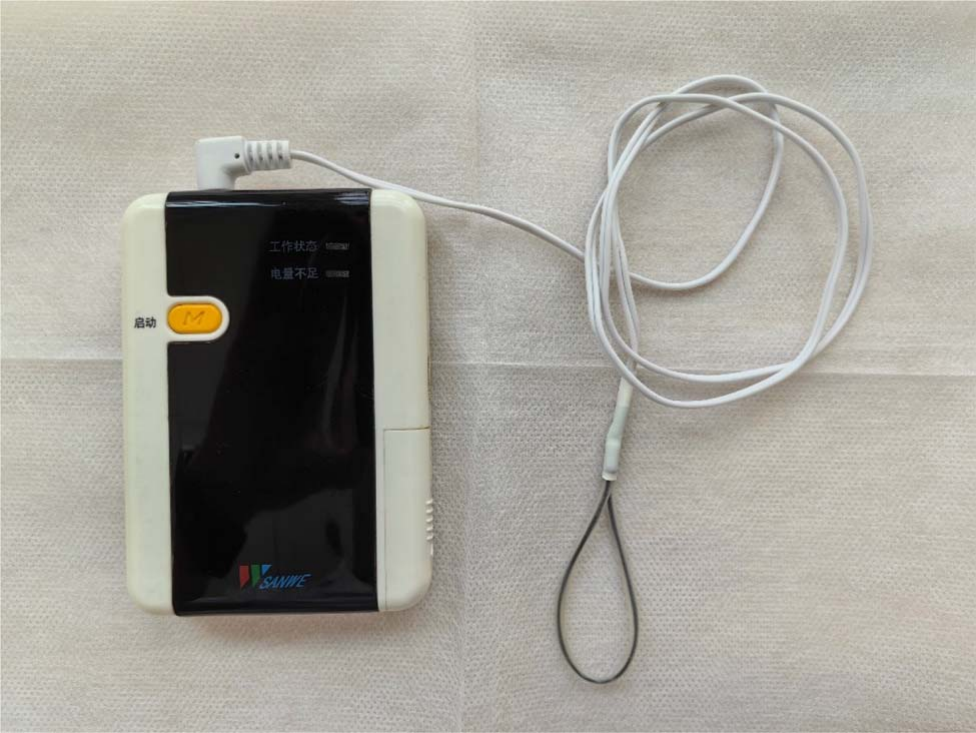
The erection recorder device.

The penile erectile function detection test was performed using a male night erection recorder device (3D Medical Technology Jiangsu Co., Ltd., MapleTiger SW-3620 Male Night Erection Recorder) (Fig. 1). Participants must meet the following requirements before testing: 1) abstinence from alcohol consumption; 2) abstinence from consumption of sleeping drugs or muscle relaxants before going to bed; 3) abstinence from consumption of stimulant beverages, such as coffee, tea, and cocoa, after 3 pm; 4) emptying of the bladder and bowels before going to bed; and 5) abstinence from sex.

### Detection method

The subjects were informed of the above precautions one day before the monitoring test, and the subjects were instructed to wear loose and comfortable clothing before the official start. First, the disposable sensor was connected to the recorder interface and the recorder power switch was turned on. The recorder box was placed face up, the strap was removed and passed through the fixed buckle, and the strap was tightened on the inner side of the thigh with moderate elasticity. Before going to bed, the penis was sterilized with iodine, and the elastic loop of the sensor was wrapped around the coronal groove of the penis, with the tightness being adequate to remain attached in the nonerect state without causing the subject to have an apparent sense of pressure. The sensor was fixed to the penis with medical tape. All data were uploaded to a computer for analysis after at least 8 h of monitoring, and an erectile event was considered if penile dilation increased girth by more than 20% for 3 min or more. In this experiment, an erection with a penile hardness greater than 60% was considered indicative of practical erectile function.

### Sex hormone and trace element measurements

Hormonal testing was performed for all patients, and specimens were always collecting in the morning. Abnormal results were repeated for confirmation. Total testosterone (TT) and estradiol (E2) were detected by fluoroimmunoassay using kits. Trace element levels were measured using atomic absorption spectrometry.

### Sperm sample collection and analysis

Before sperm extraction, the patients were required to abstain from sex for 2 to 7 days, followed by masturbation to obtain semen. Semen samples were collected in sterile sperm collection cups and immediately placed in a constant temperature incubator at 37°C for liquefaction. Semen volume was assessed with a graduation of 0.1 ml by a wide-bore graduated pipette after liquefaction. Semen analysis was performed by two blinded trained specialists according to the 2010 WHO criteria^18^.

### Surgical technique of LSG

LSG was performed through a laparoscopic approach, through devascularization of the greater curvature, from a point 2 cm proximal to the pylorus up to the His angle. With the use of linear cutting staplers, a gastric tube was made and calibrated with a 32F bougie to obtain an internal lumen of ~3 cm. During the surgical procedure, we routinely reinforce the margins, followed by an insufflation examination using a gastroscope.

### Statistical analysis

All data were analyzed using the statistical software IBM SPSS Statistics 26.0. For continuous variables, the Shapiro–Wilk test was used to test for normality. The data conforming to the normal distribution were statistically described as the mean±SD, and the categorical variables were described by frequency (percentage). Statistical inference was performed using repeated-measures ANOVA, and Bonferroni correction was used to identify the differences between time points. The *χ*^2^ test was used for categorical variables. Pearson’s correlation coefficient was used to evaluate the relationship between continuous variables. All tests were two-tailed, and *P*<0.05 was considered indicative of statistical significance. The sample size estimation was conducted using PASS 2019 software. Based on preliminary pilot study results, a statistical power of 90%, a significance level of 0.05, a dropout rate of 20%, and a two-sided paired *t*-test were employed for calculation. Ultimately, a sample size of 30 cases was determined.

## Results

This study enrolled a total of 38 patients, among whom four individuals chose to withdraw from the study due to personal reasons at the 3 months postoperative assessment. Consequently, the final cohort for analysis consisted of 34 patients. All 34 patients were followed up at 3, 6, and 12 months postoperatively. The patients’ age, BMI, marital status, and degree of education are shown in Table 1; 52.9% of the patients were between 20 and 25 years old, the proportion of BMI between 32.5 and 37.5 kg/m^2^ was 67.6%, and the largest proportions for marital status and degree of education were 67.6% single and 76.5% with a college degree or above, respectively (Table 1). All patients did not have type 2 diabetes at baseline, but among them, 18 patients were prediabetes, 26 patients had hypertension, and 23 cases had hyperlipidemia. All patients underwent successful surgery without severe postoperative complications or mortality.

**Table 1:** Baseline characteristics of study subjects.

	Number (*n*,%)
Age	
20–25	18 (52.9)
25–30	11 (32.4)
30–35	5 (14.7)
BMI	
32.5–37.5	23 (67.6)
≥37.5	11 (32.4)
Comorbidity	
Type 2 diabetes	0
Prediabetes	18 (52.9)
Hypertension	26 (76.5)
Hyperlipidemia	23 (67.6)
Marital status	
Married	9 (26.5)
Divorced	2 (5.9)
Bachelor	23 (67.6)
Degree of education	
College degree or above	26 (76.5)
Junior college or below	8 (23.5)

Prediabetes: impaired fasting glucose and/or impaired glucose tolerance.

### Anthropometric and glucolipid metabolism parameters

For anthropometric parameters, BMI significantly decreased at each follow-up point compared with baseline after LSG. The BMI decreased from 37.42±3.64 kg/m^2^ at baseline to 28.21±3.43 kg/m^2^ at 1 year, and there were significant differences between 3, 6, and 12 months after LSG. For glucolipid metabolism parameters, triglycerides, cholesterol, and low-density cholesterol were significantly decreased at 3, 6, and 12 months after surgery compared with baseline. High-density lipoprotein cholesterol increased significantly at 3, 6, and 12 months after LSG compared with preoperative levels. HOMA-IR significantly decreased at each follow-up point compared with baseline (Table 2).

**Table 2:** Comparison of the BMI and lipid variables in patients at baseline and follow-up after LSG.

	Baseline	3 months	6 months	12 months
BMI (kg/m^2^)	37.42±3.64	31.33±3.04^a^	29.58±2.78^a^ ^b^	28.21±3.43^a^ ^b^ ^c^
TC (mmol/l)	4.37±1.23	4.11±1.29^a^	4.09±1.34^a^ ^b^	4.10±1.22^a^
TG (mmol/l)	2.57±1.21	1.98±0.81^a^	1.87±1.02^a^ ^b^	1.86±1.09^b^
LDL (mmol/l)	2.52±1.04	2.39±1.07^a^	2.35±1.11^a^	2.33±1.23^a^
HDL (mmol/l)	1.14±0.38	1.18±0.31^a^	1.21±0.28^a^	1.19±0.36^a^
HOMA-IR	9.75±1.44	7.35±1.35^a^	6.81±1.27^a^ ^b^	5.39±1.17^a^ ^b^ ^c^

a *P*<0.05 on post-hoc testing between baseline and 3 months or 6 months or 12 months postoperatively.

b *P*<0.05 on post-hoc testing between 3 months and 6 months or 12 months postoperatively.

c *P*<0.05 on post-hoc testing between 6 months and 12 months postoperatively.

HDL, high-density cholesterol; HOMA-IR, homeostasis model assessment of insulin resistance; LDL, low-density cholesterol; TC, cholesterol; TG, triglycerides.

### Erectile function

Table 3 shows the IIEF-5 score, number of erections, erection hardness, and average erection duration at baseline and 3, 6, and 12 months after LSG. All of the above measures showed an upward trend after LSG and had significant differences from baseline.

**Table 3:** Comparison of erectile function in patients at baseline and follow-up after LSG.

	Baseline	3 months	6 months	12 months
IIEF-5 score	18.88±5.97	22.54±4.29^a^	23.37±3.55^a^ ^b^	23.78±3.19^a^ ^b^ ^c^
Number of erections	2.14±0.88	2.31±0.75^a^	2.65±0.83^a^ ^b^	3.05±0.96^a^ ^b^ ^c^
Erection hardness (%)	66.25±11.07	70.23±10.29^a^	73.49±9.58^a^ ^b^	78.36±9.77^a^ ^b^ ^c^
Average duration (min)	2.81±1.81	3.22±1.19^a^	3.31±1.05^a^	3.33±1.21^a^ ^b^

a *P*<0.05 on post-hoc testing between baseline and 3 months or 6 months or 12 months postoperatively.

b *P*<0.05 on post-hoc testing between 3 months and 6 months or 12 months postoperatively.

c *P*<0.05 on post hoc testing between 6 months and 12 months postoperatively.

### Sperm parameters

**Figure 2:**
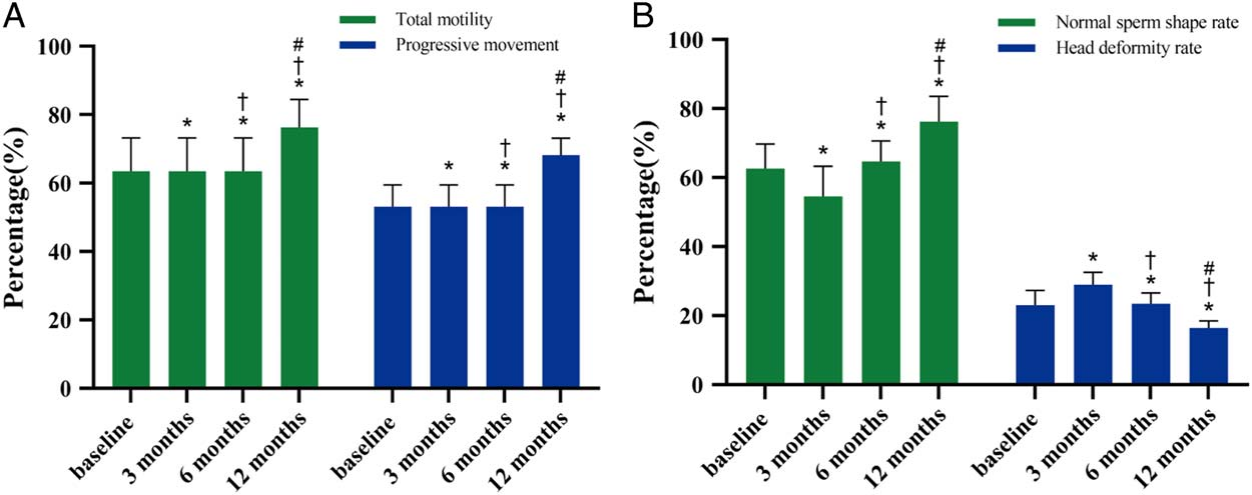
Sperm quality variables in patients at baseline and follow-up. (A) Changes of total motility and progressive movement of sperm at different times (B) Changes of normal sperm shape and head deformity rate at different times *P<0.05 on post-hoc testing between baseline and 3 months or 6 months or 12 months postoperatively †*P*<0.05 on post-hoc testing between 3 months and 6 months or 12 months postoperatively #*P*<0.05 on post-hoc testing between 6 months and 12 months postoperatively.

Abstinence duration did not differ significantly between baseline and postoperative periods. Semen volume showed a significant upward trend, from 3.28±0.78 ml at baseline to 3.43±1.13 ml at 12 months. At 3 months after LSG, the relevant seminal parameters, including sperm concentration, sperm survival rate, total motility, progressive movement, and sperm morphology parameters, decreased compared with baseline (*P*<0.05). There was a significant improvement from baseline at 6 and 12 months postoperatively (Table 4). In Figure 2A, the results for total sperm motility and forward movement at various time points are illustrated. Specifically, total sperm total motility displayed an increase from 63.45±9.78% to 76.33±8.09%, while sperm progressive movement showed an increase from 53.12±6.34% to 68.23±4.87%. In Figure 2B, normal sperm shape rate showed an increase from 62.64±7.06% to 76.21±7.36%, while head deformity rate showed a decrease from 23.06±4.19% to 16.44±2.02%.

**Table 4:** Comparison of sperm quality variables in patients at baseline and follow-up after LSG.

	Baseline	3 months	6 months	12 months
Abstinence time (day)	5.91±1.75	5.84±1.35	6.32±1.41	5.82±1.57
Semen volume (ml)	3.28±0.78	3.32±1.04^a^	3.33±0.98^a^	3.43±1.13^a^
sperm concentration (10^6/ml)	70.26±21.97	55.12±10.24^a^	68.98±13.87^a^ ^b^	86.18±12.37^a^ ^b^ ^c^
Sperm survival rate (%)	67.34±12.09	54.52±14.08^a^	70.43±11.09^a^ ^b^	80.39±10.11^a^ ^b^ ^c^
NP (%)	10.33±3.19	11.20±4.26^a^	9.03±4.10^a^	8.10±4.52^a^ ^b^
NMSR (%)	9.75±2.45	12.38±3.73^a^	9.68±2.82^a^ ^b^	5.58±3.12^a^ ^b^ ^c^
MSR (%)	4.55±0.91	4.11±1.57^a^	2.12±0.71^a^ ^b^	1.77±0.84^a^ ^b^

a *P*<0.05 on post-hoc testing between baseline and 3 months or 6 months or 12 months postoperatively.

b *P*<0.05 on post-hoc testing between 3 months and 6 months or 12 months postoperatively.

c *P*<0.05 on post-hoc testing between 6 months and 12 months postoperatively.

NP, nonprogressive movement; NMSR, neck, and middle segment deformity rate; MSR, major segment deformity rate.

### Sex hormones and trace elements

**Figure 3:**
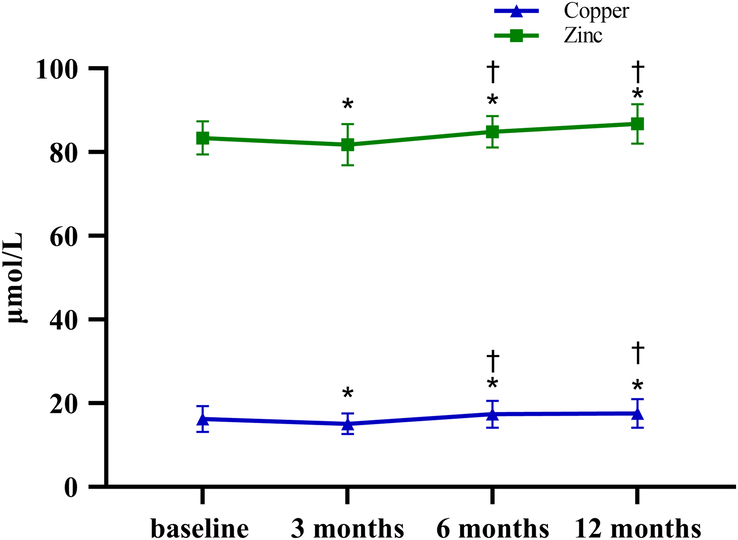
Changes of copper and zinc at different times. *P<0.05 on post-hoc testing between baseline and 3 months or 6 months or 12 months postoperatively †*P*<0.05 on post-hoc testing between 3 months and 6 months or 12 months postoperatively.

At 3 months after LSG, the levels of estradiol, iron, copper, and zinc were significantly lower compared with baseline, and the levels of testosterone were significantly increased (217.18±35.34 ng/dl vs. 415.35±50.32 ng/dl, *P* = 0.01). At 12 months postoperatively, the level of serum copper exhibited an increase, rising from 16.21±3.11 μmol/l to 17.54±3.40 μmol/l, while the levels of plasma zinc also increased from 83.40±3.97 μmol/l to 86.77±4.69 μmol/l (Fig. 3). Estradiol levels showed a downward trend compared with baseline (150.23±56.78 pmol/l vs. 121.11±30.59 pmol/l, *P* = 0.03) (Table 5).

**Table 5:** Comparison of sex hormones and trace elements in patients at baseline and follow-up after LSG.

	Baseline	3 months	6 months	12 months
TT (ng/dl)	217.18±35.34	415.35±50.32^a^	468.57±40.58^a^	512.47±48.37^a^ ^b^
E_2_ (pmol/l)	150.23±56.78	142.32±40.34	133.11±20.31	121.11±30.59^a^ ^b^
Iron (mmol/l)	8.18±0.55	8.56±1.33^a^	8.32±1.65^a^	8.42±1.38^a^

a *P*<0.05 on post-hoc testing between baseline and 3 months or 6 months or 12 months postoperatively.

b *P*<0.05 on post-hoc testing between 3 months and 6 months or 12 months postoperatively.

E_2_, estradiol; TT, total testosterone.

### Correlation analysis

Table 6 shows the correlations between baseline and postoperative 12-month BMI, sex hormones, trace elements, and HOMA-IR differences. Changes in BMI were significantly correlated with sperm concentration, semen volume, erection hardness, testosterone, estradiol, zinc, and HOMA-IR. Changes in sperm concentration were significantly correlated with changes in testosterone, estradiol, zinc, and HOMA-IR. The changes in erection hardness were significantly correlated with the changes in testosterone, estrogen, and HOMA-IR.

**Table 6:** The correlation analysis between the BMI variations and changes in the sex hormone levels, trace elements, and sperm parameters.

	ΔBMI	Δsperm concentration	Δsperm volume	ΔErection hardness	ΔTT	ΔE_2_	ΔCopper	ΔZinc	ΔHOMA-IR
ΔBMI	\	\	\	\	\	\	\	\	\
Δsperm concentration	r=0.23 *P*=0.032	\	\	\	\	\	\	\	\
Δsperm volume	r=0.15 *P*=0.045	r=0.22 *P*=0.142	\	\	\	\	\	\	\
ΔErection hardness	r=0.32 *P*=0.021	r=0.15 *P*=0.352	r=0.09 *P*=0.731	\	\	\	\	\	\
ΔTT	r=0.45 *P*=0.002	r=0.43 *P*=0.013	r=0.19 *P*=0.063	r=0.33 *P*=0.007	\	\	\	\	\
ΔE_2_	r=0.23 *P*=0.032	r=0.34 *P*=0.041	r=0.25 *P*=0.071	r=0.23 *P*=0.048	r=0.27 *P*=0.331	\	\	\	\
ΔCopper	r=0.15 *P*=0.052	r=0.34 *P*=0.083	r=0.29 *P*=0.059	r=0.28 *P*=0.251	r=0.08 *P*=0.451	r=0.11 *P*=0.349	\	\	\
ΔZinc	r=0.33 *P*=0.004	r=0.25 *P*=0.033	r=0.31 *P*=0.016	r=0.14 *P*=0.098	r=0.08 *P*=0.273	r=0.04 *P*=0.381	r=0.11 *P*=0.349	\	\
ΔHOMA-IR	r=0.35 *P*=0.001	r=0.23 *P*=0.026	r=0.12 *P*=0.631	r=0.43 *P*=0.033	r=0.17 *P*=0.051	r=0.11 *P*=0.231	r=0.21 *P*=0.141	r=0.16 *P*=0.111	\

Variations (Δ) in BMI and HOMA-IR are reported between baseline and 12 months, and changes in the levels of erectile function, sex hormones, sperm concentration, and sperm volume were assessed (Δ between 12 months and baseline).

E_2_, estradiol; HOMA-IR, homeostasis model assessment of insulin resistance; TT, total testosterone.

## Discussion

Obesity is known to significantly elevate the risk of various diseases and negatively impact mental health^19^. Moreover, the influence of obesity on male reproductive function is becoming more evident, as it can contribute to male reproductive disorders, including ED and reduced semen quality^20,21^. This study observed significant improvements in BMI, blood lipids, and other indicators at 3, 6, and 12 months following LSG. The patients’ BMI decreased from 37.42±3.64 kg/m^2^ at baseline to 28.21±3.43 kg/m^2^ at 12 months postsurgery, indicating a reduction in visceral fat. Severe obesity is associated with insulin resistance, which is linked to cardiovascular risk markers such as hypertriglyceridemia and lipoprotein disorders^22^. Insulin resistance improved from the preoperative stage to 12 months postoperatively (9.75±1.44 vs. 5.39±1.17, *P*<0.05). Previous studies have demonstrated that insulin resistance interacts with glucose metabolism and lipid metabolism^23,24^. The remission of insulin resistance following bariatric surgery can significantly improve systemic metabolic status^25^. Additionally, a meta-analysis of randomized controlled trials comparing weight loss and nonweight loss interventions indicated that patients who underwent bariatric surgery experienced greater improvements in glucose homeostasis, weight loss, plasma triglyceride levels, and high-density lipoprotein cholesterol^26^. These findings align with our results, which demonstrated improvements in BMI, blood lipids, and HOMA-IR after LSG, thereby promoting overall metabolic health.

This study demonstrated that the postoperative IIEF-5 score increased from 18.88±5.97 (preoperatively) to 23.78±3.19 (at 12 months postoperatively), indicating improvement in erectile function. Additionally, the hardness of the erections increased from 66.25±11.07% to 78.36±9.77%. Furthermore, the number of erections and the average duration of erections were significantly higher at 12 months postoperatively compared to baseline. These findings are consistent with a study conducted by Liu *et al*.^27^ showed that the brief male sexual function inventory score was also significantly increased after bariatric surgery. Compared with their studies, this study more fully confirmed the improvement of erectile function by bariatric surgery from the IIEF-5 score and multiple indicators of night-time erectile function detection.

Penile erection is a multifaceted process that involves the regulation of vascular, endocrine, and neural factors. Pathological alterations in the penile vascular endothelium can contribute to ED^28^. In China, central obesity characterized by the accumulation of visceral fat is prevalent. Compensatory hyperinsulinemia and abnormal levels of circulating factors originating from adipose tissue contribute to the development of atherosclerosis and a reduction in endothelium-dependent vasodilation, thereby directly impacting male sexual function, particularly the occurrence of ED^29^. Insulin resistance has been confirmed as a risk factor for ED^30^. Consistently, our study also established a notable improvement in insulin resistance following LSG. Additionally, the correlation analysis revealed a significant association between penile erection hardness and the improvement of insulin resistance (r=0.43, *P*=0.033). These findings suggest that the amelioration of erectile function following LSG may be linked to the remission of insulin resistance.

Obesity is known to be associated with decreased plasma total testosterone levels in men, which increases the risk of vascular pathology and hypogonadism^31^. Furthermore, studies conducted by Kun *et al*.^9^ have demonstrated that improvements in cavernous tissue morphology, vascular disease, and changes in the carotid artery wall are associated with enhancements in erectile function. The results of our study also indicated a continuous increase in total testosterone levels following LSG, and there was a significant correlation between testosterone levels and erectile function (r=0.33, *P*=0.007). Testosterone replacement therapy is commonly utilized in ED patients with hypogonadism^32^. This further suggests that the rise in testosterone levels after LSG is also a significant contributing factor to the improvement in erectile function.

An intriguing observation regarding semen quality was made in this study. Overall, there was a significant decrease in sperm concentration, sperm survival rate, total motility, and sperm morphology parameters at 3 months postoperatively compared to baseline. However, these parameters showed a significant increase at 6 and 12 months postoperatively (Table 4, Fig. 2). Specifically, the sperm concentration decreased from 70.26±21.97 10^6/ml to 55.12±10.24 10^6^/ml at 3 months and then increased to 86.18±12.37 10^6/ml at 12 months. These findings indicate that semen quality did not improve simultaneously with erectile function during the initial 3 months following surgery. However, indicators related to semen quality demonstrated improvement at 6 and 12 months postsurgery compared to baseline and 3 months postsurgery. The study performed by Abouelgreed *et al*. shown that the sperm count increased at 12 and 18 months after LSG, but the sperm motility did not significantly improve. The results of the early 3 and 6 months after LSG did not explain, and also changes in trace elements in the body were not described^33^. These results suggest that multiple factors may influence semen quality after LSG.

Considering that all patients included in this study underwent LSG and were nonsmokers and nondrinkers prior to the operation. The decline observed in semen quality, especially in sperm concentration, total motility, survival rate, and sperm morphology within 3 months postsurgery, is hypothesized to have two primary reasons: Firstly, the dietary restrictions within the initial 3 months postsurgery, resulting in reduced protein intake and deficiencies in micronutrients^34,35^. Secondly, the release of lipophilic endocrine-disrupting chemicals postsurgery, which collectively impacts the three main stages of spermatogenesis: proliferation of spermatogonia, meiotic division of spermatocytes, and alterations in sperm shape and nuclear content, ultimately affecting sperm concentration and morphology^36,37^. The rapid weight loss causing intense metabolic changes, particularly lipophilic endocrine-disrupting chemicals, decreases semen quality. However, this decline is reversible once weight stabilizes. As dietary intake normalizes and nutritional supplementation occurs, particularly with metabolic improvements due to weight loss, there is a significant positive impact on long-term semen quality improvement.

An observation was made that preoperative obese patients exhibited elevated levels of estradiol and significantly lower levels of total testosterone. However, following the surgery, there was a substantial decrease in estradiol levels and a significant increase in testosterone levels compared to baseline. Existing evidence supports the notion that bariatric surgery can gradually restore sex hormone levels to normal^11^. Lee *et al*.^38^ (2019) concluded that testosterone increased while estradiol decreased after bariatric surgery. Furthermore, the present study identified a correlation between changes in sperm concentration, testosterone, and estradiol, highlighting the influence of sex hormones on semen quality.

Previous studies have consistently reported a close relationship between zinc and spermatogenesis^39,40^. The deficiency of trace elements following bariatric surgery is a significant concern, and it has been reported that the incidence of zinc deficiency after LSG and RYGB surgery ranges from 7 to 15% and 20 to 37%, respectively^41^. Our study demonstrated a significant decline in zinc and copper levels at 3 months postoperatively. However, there was a subsequent increase in zinc and copper levels at 6 and 12 months postoperatively, coinciding with the improvement in semen quality after LSG. Furthermore, correlation analysis revealed a significant association between changes in zinc levels and semen quality, indicating the impact of zinc on semen quality. Similarly, a study conducted by Berniza *et al*.^42^ indicated that reproductive hormones tend to reach normal levels after bariatric surgery. However, the concentrations of zinc and copper decreased, and sperm quality did not significantly improve. Many studies have confirmed that supplementing nutrients such as zinc, copper, folic acid, and vitamin B12 has a positive impact on improving sperm quality^43^. Specifically, zinc and copper play crucial roles in sperm motility, energy acquisition, and acrosome reaction, regulating the stable environment for sperm^44,45^.

Our study’s results of improved erectile function postbariatric surgery align with previous research indicating a positive association between weight reduction and enhanced sexual health. It is well-established that obesity contributes to vascular and hormonal disturbances, affecting erectile function. The positive changes observed in our study not only highlight the potential benefits for individual sexual well-being but also suggest a broader public health impact. Furthermore, our investigation into semen quality parameters, including sperm count, motility, and morphology, provides valuable insights into the reproductive potential of individuals undergoing bariatric surgery after 12 months. Understanding the interplay between bariatric surgery, erectile function, and semen quality has implications for both clinical practice and public health. Beyond individual patient outcomes, these improvements may translate into increased fertility rates and improved reproductive health at the population level. Acknowledging the broader public health implications of our results emphasizes the relevance of weight-loss interventions in addressing not only individual health concerns but also contributing to the overall reproductive well-being of the male population.

The present study has several limitations. First, the sample size of the study was relatively small, which may limit the generalizability of the findings. Further validation in a larger population is necessary to confirm the results and provide more robust evidence. Second, the study focused on evaluating the impact of LSG on early male reproductive function at 12 months postsurgery. The reproductive status of male patients after LSG was not comprehensively assessed, and long-term outcomes beyond 12 months were not evaluated. Future studies should consider assessing the long-term effects of LSG on male reproductive function to provide a more comprehensive understanding of the topic.

## Conclusions

LSG has been shown to significantly improve BMI, blood lipids, and insulin resistance in Chinese men with obesity. Additionally, it has a positive impact on erectile function. However, it should be noted that semen quality experiences a temporary decline at 3 months after LSG. This decline is observed in parameters such as sperm concentration, total motility, survival rate, and sperm morphology. Nevertheless, semen quality gradually improved at 6 and 12 months after LSG.

## Ethical approval

The study was approved by the Institutional Review Board at Third Xiangya Hospital of Central South University. Approval No: R19046.

## Consent

Written informed consent was obtained from the patient for publication and any accompanying images. A copy of the written consent is available for review by the Editor-in-Chief of this journal on request.

## Sources of funding

This study was supported by the Wisdom Accumulation and Talent Cultivation Project of the Third Xiangya Hospital of Central South University [YX202106].

## Author contribution

X.G.: conceptualization, methodology, formal analysis, data curation, and writing – original draft; P.L.: conceptualization, methodology, data curation, and writing – original draft; G.W. and W.L.: formal analysis and data curation; Z.S.: validation, visualization, and writing – original draft; L.Z. and S.Z.: writing review and editing and supervision.

## Conflicts of interest disclosure

There are no conflicts of interest.

## Research registration unique identifying number (UIN)

1. Name of the registry: ClinicalTrials.gov.
2. Unique identifying number or registration ID: NCT04237311.
3. Hyperlink to your specific registration (must be publicly accessible and will be checked): https://clinicaltrials.gov/study/NCT04237311?id=NCT04237311.&rank=1.

## Guarantor

Shaihong Zhu, M.D., Department of General Surgery, Third Xiangya Hospital, Central South University, 138 Tongzipo Street, Changsha 410013, Hunan, People’s Republic of China.

## Data availability statement

The datasets generated during the current study are available from the corresponding author on reasonable request.

## Provenance and peer review

Not commissioned, externally peer-reviewed.
